# Commentary: What is the key issue in high take-off of the left coronary artery?

**DOI:** 10.1016/j.xjtc.2021.06.020

**Published:** 2021-06-23

**Authors:** Junjiro Kobayashi

**Affiliations:** Department of Cardiovascular Surgery, National Cerebral and Cardiovascular Center, Suita, Osaka, Japan

**Central Message**

Keys issues in patients with high take-off of the LCA are myocardial protection during surgery and potential risk of sudden death due to coronary ostial stenosis.

**Figure:**
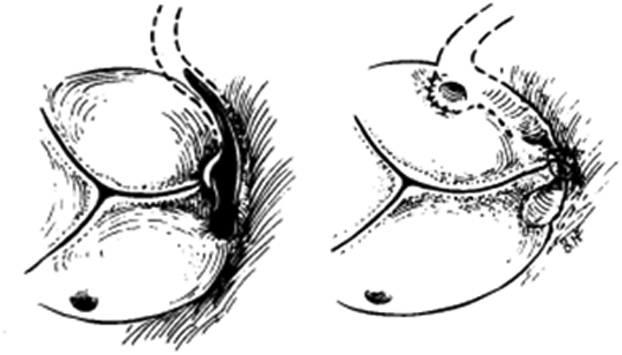
Surgical technique for anomalous origin of the LCA from the right coronary sinus.

See Article page 53.

Iacona and colleagues^1^ present an interesting case report on a very rare case of high take-off of the left coronary artery. The patient underwent coronary artery bypass grafting, aortic valve replacement, and aortoplasty. The authors emphasize the importance of preoperative identification of the exact origin of the left circumflex artery (LCX) and surgical planning.

Fortunately, there was space to clamp the aorta between the aortic cannulation site and the origin of the LCX in this patient. Therefore, a standard operation was possible if the LCX was identified and not damaged. The method of myocardial protection is cumbersome when aortic crossclamp is not possible distal to the origin of the LCX. In this situation, circulating blood is continuously supplying the left ventricle muscle, and myocardial protection is not optimal. This situation is similar to redo cardiac operation when the left internal thoracic artery to the left anterior descending artery bypass is present. Options have been suggested, such as deep hypothermia without circulatory arrest, ventricular fibrillation with continuous antegrade coronary perfusion with or without retrograde coronary perfusion, and clamping of the LCX with continuous retrograde cardioplegia. I recommend retrograde cardioplegic infusion and systemic hyperkalemia with moderate hypothermia^2^ because of its simplicity.

The other issue is sudden cardiac death in patients with anomalous origin of the coronary artery from the wrong aortic sinus.^3^^,^^4^ Impaired coronary perfusion at the level of the coronary ostium has been reported. The anomalous coronary artery frequently passes between the aorta and the pulmonary artery (interarterial course) commonly running within the aortic wall (intramural course), which may cause ostial stenosis and myocardial ischemia. The natural history of anomalous origin of a coronary artery is still controversial. The combined US and Italian autopsy registry reported 27 sudden deaths in competitive athletes between ages 9 and 32 years resulting from coronary artery anomalies with wrong aortic sinus.^3^ In patients with clinical data, 83% had symptoms before sudden death. Asymptomatic patients with anomalous origin of the right coronary artery and good left ventricular function older than age 40 years had benign prognosis.^4^

**Figure 1:**
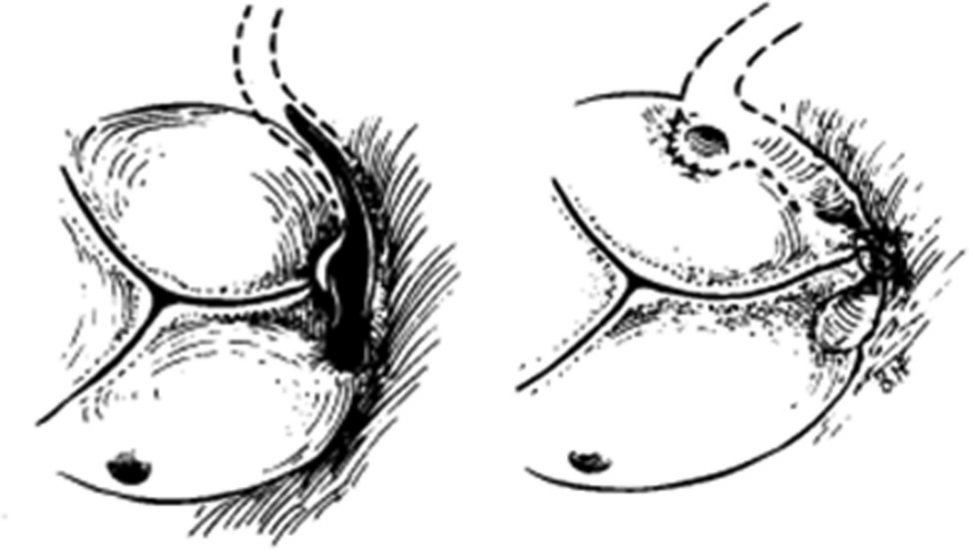
Surgical technique for anomalous origin of the left coronary artery from the right coronary sinus. The ostium was split open, and the incision was extended above the intercoronary commissure along the intramural segment of the left main coronary artery to the midpoint of the left coronary sinus. Revised from Mustafa and colleagues.^5^

In the present case, coronary artery bypass grafting to left anterior descending artery, an obtuse marginal branch, and right coronary artery was performed in addition to aortic valve replacement and aortoplasty. Therefore, a direct approach to the origin of left coronary artery is unnecessary (Figure 1).^5^ Preoperative identification of the exact origin of the LCX and evaluation of myocardial ischemia is important in elderly patients.
